# Construction of a high-density genetic map and mapping of growth related QTLs in the grass carp (*Ctenopharyngodon idellus*)

**DOI:** 10.1186/s12864-020-6730-x

**Published:** 2020-04-19

**Authors:** Xiaoli Huang, Yanxin Jiang, Wanting Zhang, Yingyin Cheng, Yaping Wang, Xiaocui Ma, You Duan, Lei Xia, Yaxin Chen, Nan Wu, Mijuan Shi, Xiao-Qin Xia

**Affiliations:** 1 Institute of Hydrobiology, the Chinese Academy of Sciences, Wuhan, China; 2 University of Chinese Academy of Sciences, Beijing, China

**Keywords:** Grass carp, Linkage map, Growth-related trait, Quantitative trait loci, Single nucleotide polymorphism

## Abstract

**Background:**

Grass carp (*Ctenopharyngodon idellus*) are important species in Asian aquaculture. A draft genome for grass carp has already been published in 2015. However, there is still a requirement for a suitable genetic linkage map to arrange scaffolds on chromosomal frameworks. QTL analysis is a powerful tool to detect key locations for quantitative traits, especially in aquaculture. There no growth related QTLs of grass carp have been published yet. Even the growth trait is one of the focuses in grass carp culture.

**Results:**

In this study, a pair of distantly related parent grass carps and their 100 six-month-old full-sib offspring were used to construct a high-density genetic map with 6429 single nucleotide polymorphisms (SNPs) by 2b-RAD technology. The total length of the consensus map is 5553.43 cM with the average marker interval of 1.92 cM. The map has a good collinearity with both the grass carp draft genome and the zebrafish genome, and it assembled 89.91% of the draft genome to a chromosomal level. Additionally, according to the growth-related traits of progenies, 30 quantitative trait loci (QTLs), including 7 for body weight, 9 for body length, 5 for body height and 9 for total length, were identified in 16 locations on 5 linkage groups. The phenotypic variance explained for these QTLs varies from 13.4 to 21.6%. Finally, 17 genes located in these regions were considered to be growth-related because they either had functional mutations predicted from the resequencing data of the parents.

**Conclusion:**

A high density genetic linkage map of grass carp was built and it assembled the draft genome to a chromosomal level. Thirty growth related QTLs were detected. After the cross analysis of Parents resequencing data, 17 candidate genes were obtained for further researches.

## Background

The grass carp (*Ctenopharyngodon idellus*) belongs to the Cyprinidae family and is the only species of the genus *Ctenopharyngodon*. As one of the most important freshwater-cultured fish, the global production of grass carp was approximately 5.8 million tons, accounting for 12.18% of global freshwater fish production in 2015 [1]. Currently most studies about grass carp focused on fish immunity [2–4], nutrition [5–7], and stress resistance [8]. A few growth-related studies in grass carp were focused on the impacts of the additives, dietary, or the growth hormones [9]. Therefore, the underlying genes associated with growth traits are still waiting to be revealed.

Growth traits are typical quantitative traits, which are influenced by multiple genes, and perhaps no any single gene shows significant impact on such a trait. So it is very difficult to discover these genes through reverse genetics, especially for the grass carp, a fish specie usually breed once a year. Forward genetic techniques, such as quantitative trait locus (QTL) location, are more effective in parsing genes for complex traits. As early as in the first few years of the new century, QTL research was applied to investigate the body weight trait in rainbow trout (*Oncorhynchus mykiss*) [10], the first economic fish with genetic linkage mapping [11]. In the same year, a QTL for body length of tilapia (*Oreochromis mossambicus* and *Oreochromis aureus*) was published [12] . Since then, deeper researches on growth-related traits also have been undertaken in other teleost fishes, such as Atlantic salmon (*Salmo salar*) [13]. In recent years, QTL studies on growth-related traits were reported in some of the main farmed species in China, e.g., common carp (*Cyprinus carpio*) [14] and bighead carp (*Hypophthalmichthys nobilis*) [15]. However, there is no similar research in the grass carp as yet.

As the current dominated molecular markers used in researches of grass carps [16–20], microsatellites (or Simple Sequence Repeats, SSRs), were not suitable for the high-throughput genotyping methods. With the technological advances, the high-throughput SNP genotyping methods, such as SNP array [21] and next-generation sequencing (NGS), have been widely applied in construction of genetic maps and location of QTLs in the teleost fishes [14, 15, 22]. The SNP calling and genotyping have become feasible in grass carp since the publication of the draft genome [23].

Earlier NGS methods for QTL analysis, including reduced representation sequencing, complexity reduction of polymorphic sequences (CRoPS), restriction site associated DNA sequencing (RAD-seq), and low coverage genotyping, have been discussed previously [24]. The prominent advantage of RAD-seq is the reduction of labor and cost, due to the pooled library. However, possible genotyping errors, caused by many factors [25], have been already revealed for RAD-seq. An improved method, 2b-RAD, avoids most of potential errors which may come out of size selection or sequencing depth. Furthermore, 2b-RAD is suitable for parallel genotyping for more samples, and can be more flexible with adjusting the marker density [26].

Comparatively, linkage maps have provided a framework for genomic and genetic studies, such as molecular marker-assisted selection (MAS) for quality [27] and quantitative traits (QTL), as well as chromosomal frameworks for genome scaffolds. The genetic maps for many aquaculture species have been published [14, 15, 28–31]. A high-density genetic linkage map can provide a more precise localization of the loci related to target traits and mount more genomic contigs. The first genetic linkage map of grass carp has a low density with 279 markers [32]. A map with a high density is urgently needed for the genome frameworks and the locations of trait-related loci and genes.

In this study, a high-density genetic linkage map was constructed as a chromosome framework for the draft genome assembling and it mounted 89.91% of the genome sequences, much higher than the mounting rate (64%) of the first genetic map. Thirty QTL loci for growth-related traits were then located on the map and a candidate gene list for subsequent growth research was obtained.

## Results

### SNP marker filtration

The genomic high-throughput sequencing data from 2 parents and 100 progenies was screened by SOAP2 [33] and RADtyping software [34]. As a result, 5818 codominate markers (SNP) and 3531 dominate markers (InDel), belonging to 16,359 tags were preliminarily selected. After further filtration using the software bowtie and bowtie 2, a total of 8608 truly unique tags were obtained.

After excluding makers with significant segregation distortion (*χ*^2^ test, *p* < 0.05, df = 2), 6658 markers on 6602 tags were obtained for constructing the genetic linkage map. The average interval between markers in the genome was 0.119 Mb. The markers were distributed over 610 supercontigs, which covered 93.47% of the grass carp draft genome. In order to construct genetic linkage maps quickly and accurately, the location markers that were identical were merged, which allowed for the absence of missing genotypes in offsprings, due to the limitations of joinmap 4.1 regarding the number of makers. Then the markers with the highest number of successful genotypes in offspring were used as representative markers for constructing the map. From all the markers, 3381 markers were divided into 767 groups, and 3099 markers were not consistent with any others. 122 markers were ambiguous because they could be divided into two or more different groups due to missing genotypes. Therefore, 3866 actual markers (a-markers) for the map were obtained after ambiguous markers were removed.

### The construction of the linkage map

The 3866 a-markers and all related SNPs were divided into 24 groups, with LOD ≥ 5.0. The ML algorithm in Joinmap 4.1 and the Mergemap [35] were used to construct the linkage maps of both the parents (male or female) and consensus. The result showed that the male map were consisted of 3875 markers distributed in 1973 loci with a total length of 6301.59 cM and an average interval of 3.23 cM (Supplementary Table S1). The female map consisted of 3742 markers distributed in 1898 loci with a total length of 5680.51 cM and an average interval of 2.89 cM (Supplementary Table S1). The consensus map consisted of 6429 SNPs distributed in 3340 loci with a total length of 5553.43 cM and an average interval of 1.92 cM (Table 1). Since the ML distance was longer than the regression one, the length of linkage groups obtained were generally longer than that of other maps generated throughthe regression algorithm [14, 15, 36, 37], included the first grass carp genetic maps [32].

**Table 1:** Summary statistics of the sex-averaged linkage map of grass carp

LG name	Num of SNPs	Num of loci	LG length (cM)	Average Dist (cM)
Group1	286	150	274.303	1.841
Group2	219	135	232.437	1.735
Group3	277	132	229.354	1.751
Group4	232	131	226.073	1.739
Group5	468	287	671	2.346
Group6	231	95	149.552	1.591
Group7	261	140	216.892	1.56
Group8	204	95	181.916	1.935
Group9	264	123	224.741	1.842
Group10	187	88	205.308	2.36
Group11	352	171	319.013	1.877
Group12	331	175	318.608	1.831
Group13	344	168	341.467	2.045
Group14	316	157	304.876	1.954
Group15	255	163	364.771	2.252
Group16	274	146	285.544	1.969
Group17	298	146	257.376	1.775
Group18	238	137	314.903	2.315
Group19	287	127	244.169	1.938
Group20	231	123	219.688	1.801
Group21	229	118	210.348	1.798
Group22	224	117	211.94	1.827
Group23	208	115	220.149	1.931
Group24	213	101	149.473	1.495

*LG* linkage group, *cM* centiMorgan

From the total number of markers in maps of female, male and consensus, only 1188 markers were found as the hereozygous loci in both parents (ab × ab). These markers accounting for 18.47% of all markers. Therefore, the total number of SNPs on the concensus map far exceeded male or female maps. This finding confirmed that the parents selected in our research were indeed very different and their offspings were suitable for constructing genetic maps (pseudo-testcross).

### Genome scaffold anchoring and synteny analysis of zebrafish

**Fig. 1:**
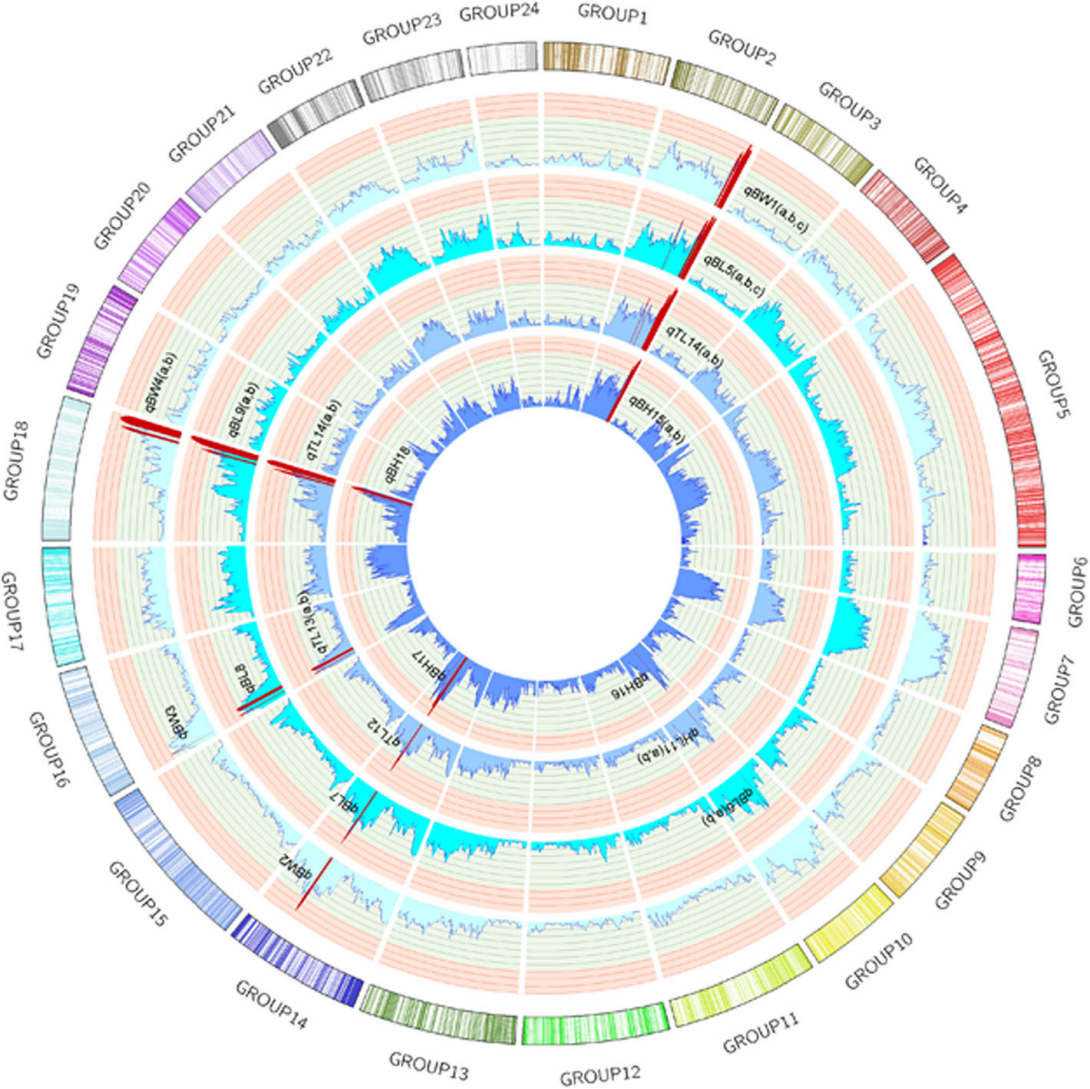
The concensus genetic map and growth-related QTLs of the grass carp. The outmoset circle was the concensus genetic map. The circles inside showed the LOD score of each markers to the four growth-related traits. The order was BW,BL,TL and BH inwardly. The QTLs were marked by dark red

All 6429 markers were distributed on 605 supercontigs with a total length of 0.81 Gb, 89.91% of the grass carp draft genome. The 99 supercontigs with more than 20 SNPs, were selected for scaffold anchoring, and they also showed a good linear relationship with the linkage groups (Fig. 1a). The 99 supercontigs, covering 642 Mb (74.39%) of the total length, were longer than the 573 Mb used previously [23]. In addition, 45 of this supercontigs were reversed.

A similar strategy for screening unique tags has been applied to map SNP tags to the zebrafish genome. Consequently, 511 unique tags were obtained. Among these unique tags, 506 fell on zebrafish chromosomes. These SNPs showed a good macro-collinearity between grass carp and zebrafish (Fig. 1b). The factor that LG13 was syntenic to ZF10 (NC_007121.6) and ZF22 (NC_007133.6) was consistent with previous results [32].

### QTL mapping of growth-related traits

Pairwise comparisons were conducted among the four growth-related traits (TL, BL, BH, BW), using Pearson’s correlation coefficient. It was revealed that all of the traits showed a high correlation (*p* < 2.2e-16). The correlation coefficients of BW/BL, BW/TL and BW/BH were 0.95, 0.95 and 0.93, respectively. The highest coefficient was 0.97 between BL and TL, and the lowest was 0.89 between BL and BH (Table S2). BL and TL conformed to the normal distribution (*p*_BL_ = 0.175, *p*_TL_ = 0.550) and the logarithm of BW and BH also conformed to the normal distribution (*p*_log(BW)_ = 0.274, *p*_log(BH)_ = 0.096).

**Fig. 2:**
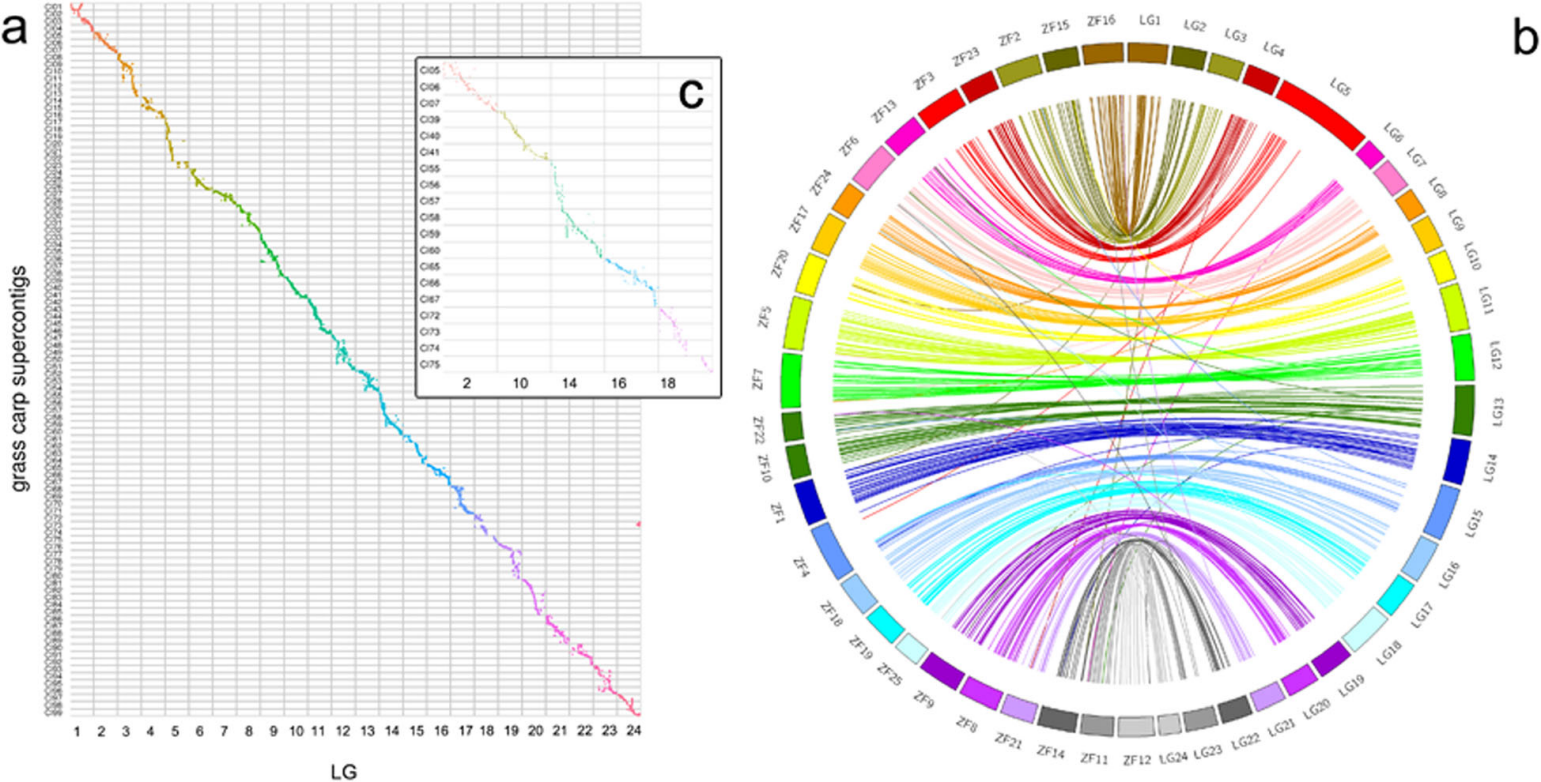
The colinearity of the genetic maps with the genome of grass carp and zebrafish. **a** The colinearity of the LGs to the 99 supercontigs in the genome of grass carp. **b**. The synteny analysis result of the LGs to zebrafish genome. **c**. The five LGs which were located growth-related QTLs

Based on the above treated phenotype data, 30 growth-related QTLs were found on 10 genome regions (GRs), 16 genetic linkage regions (GLRs), or five LGs including LG2, LG10, LG14, LG16 and LG18 (Table 2, Fig. 2). These LGs and corresponding supercontigs were in the synteny (Fig. 1c).

**Table 2:** The statistics of QTLs

Traits	QTL name	LG	Position (cM)	LOD	Exp%	St. stie on Draft		End site on Draft		Nearest marker
						supercontigs	site	supercontigs	site	
BW	qBW1a	2	214.34	4.39	18.3	CI01000095	529,014	CI01000095	531,012	ref-59,824
	qBW1b	2	223.359	5.1	20.9	CI01000093	649,736	CI01000093	1,143,791	ref-37,413
	qBW1c	2	229.268	4.72	19.5	CI01000093	429,749	CI01000093	662,875	ref-10,619
	qBW2	14	220.328	4.61	19.1	CI01000300	4,435,910	CI01000300	4,575,906	ref-99,718
	qBW3	16	12.892	3.52	15.0	CI01000025	235,941	CI01000025	253,860	ref-105,583
	qBW4a	18	305.478	4.48	18.6	CI01000142	246,876	CI01000153	714,348	ref-27,615
	qBW4b	18	284.534	4.27	17.8	CI01000123	278,805	CI01000123	285,480	ref-111,308
BL	qBL5a	2	214.34	4.16	17.4	CI01000095	529,014	CI01000095	531,012	ref-59,824
	qBL5b	2	222.359	4.64	19.2	CI01000093	649,736	CI01000093	1,143,791	ref-37,413
	qBL5c	2	229.268	4.54	18.9	CI01000093	429,749	CI01000093	662,875	ref-10,619
	qBL6a	10	70.103	3.13	13.4	CI01000051	4,515,076	CI01000051	5,243,968	ref-38,239
	qBL6b	10	70.161	3.13	13.4	CI01000051	4,515,076	CI01000051	5,243,968	ref-38,239
	qBL7	14	220.328	4.13	17.3	CI01000300	4,435,910	CI01000300	4,575,906	ref-99,718
	qBL8	16	12.892	3.37	14.4	CI01000025	235,941	CI01000025	253,860	ref-105,583
	qBL9a	18	310.478	4.58	19.0	CI01000142	246,876	CI01000153	714,348	ref-91,024
	qBL9b	18	284.534	3.7	15.7	CI01000123	278,805	CI01000123	285,480	ref-111,308
TL	qTL10a	2	214.34	4.59	19.1	CI01000095	529,014	CI01000095	531,012	ref-59,824
	qTL10b	2	223.359	5.25	21.5	CI01000093	649,736	CI01000093	1,143,791	ref-37,413
	qTL11a	10	70.103	3.23	13.8	CI01000051	4,515,076	CI01000051	5,243,968	ref-38,239
	qTL11b	10	70.161	3.23	13.8	CI01000051	4,515,076	CI01000051	5,243,968	ref-38,239
	qTL12	14	220.328	4.17	17.5	CI01000300	4,435,910	CI01000300	4,575,906	ref-99,718
	qTL13a	16	32.888	3.49	14.8	CI01000165	610,647	CI01000166	419,046	ref-134,757
	qTL13b	16	33.551	3.49	14.8	CI01000165	610,647	CI01000166	419,046	ref-134,757
	qTL14a	18	284.534	4.62	19.2	CI01000123	278,805	CI01000123	285,480	ref-111,308
	qTL14b	18	304.478	5.28	21.6	CI01000142	246,876	CI01000153	714,348	ref-27,615
BH	qBH15a	2	223.359	4.12	17.3	CI01000093	649,736	CI01000093	1,143,791	ref-37,413
	qBH15b	2	229.268	4.26	17.8	CI01000093	429,749	CI01000093	662,875	ref-10,619
	qBH16	10	81.015	3.23	13.8	CI01000325	439,637	CI01000325	468,239	ref-178,556
	qBH17	14	220.328	4.26	17.8	CI01000300	4,435,910	CI01000300	4,575,906	ref-99,718
	qBH18	18	300.478	3.79	16.0	CI01000142	246,876	CI01000153	714,348	ref-27,615

For BW, there were 7 QTL regions, among which QTL qBW1b was with the highest LOD 5.1. QTL qBW1b was located on LG2 (at 221.359–226.697 cM), which accounted for 20.9% of the phenotypic variance (PVE). 9 QTL regions were identified for BL, and QTL qBL5b showed the highest LOD 4.64. QTL qBL5b was located on the same place as QTL qBW1b, and it accounted for 19.2% of the PVE. For TL, 9 QTL regions were identified. The correlation between TL and BL traits was the highest, the highest LOD of QTL regions of TL and BL were slightly different. The QTL with the highest LOD value was qTL14b, which was located on LG18 (at 298.478–314.903 cM), accounting for 21.6% of the PVE. For BH, five QTL regions were found. The highest LOD (4.26) was found for QTL qBH15b and qBH 17, which explained 17.8% of the PVE.

### Candidate gene identification for growth-related traits

In order to detect candidate genes more accurately, the parental whole genomes were resequenced with an average depth of 30X. A total of 2,415,558 SNPs were revealed as heterozygous for at least one parent. The main genotypes were ab × aa, aa × ab and ab × ab, which accounted for 39.19, 38.99 and 21.70% of all SNPs, respectively, while the rest SNPs (0.13%) havethree or more genotypes in parents (Table S3). In addition, 1,135,559 InDels were obtained.

Since 30 QTLs located on 16 GLRs or 10 GRs (Table 2), the responsible regions were used to scan the candidate genes. The start and end sites of the GRs were makerd by the QTL adjacent SNPs. The endpoints of the eight GRs were located on the same supercontigs, such as qBW1a (Table 2). For these GRs, genes located within the interval were extracted. Whereas, for the other two GRs, the state and end sites were located on different short supercontigs, eg. qBW4a (Table 2). For these two GRs, all genes were extracted. As the result, 49 pre-candidate genes were discovered. The further filter criterion was retained the genes which had at least one functional SNPs/InDels and finally 17 candidate genes were selected (Table 3).

**Table 3:** The Statistic of Growth-related candidate genes with mutations

QTL	Gene symbl	Gene ID	Total mutations		Functional mutations	
			SNPs	Indels	SNPs	Indels
qBL6a,qTL11a	*flrt2*	CI01000051_04796878_04799272	24	23	2	0
qBL6b,qTL11b	*serpina1*	CI01000051_05048070_05049768	7	20	0	0
qBH16	*snx14*	CI01000325_00423661_00470758	83	61	4	2
qBW2,qBL7,qTL12,qBH17	*dlc1*	CI01000300_04581207_04605807	75	70	6	0
qBW3,qBL8	*prtga*	CI01000025_00216623_00242426	143	113	9	0
qTL13a	*thsd4*	CI01000166_00408136_00415727	74	16	5	1
qTL13b	*celf6*	CI01000165_00657050_00657275	39	19	1	0
qBW4b,qBL9b,qTL14a	*adamts20*	CI01000123_00321346_00376894	184	94	8	1
qBH18	*zmym4*	CI01000142_00345504_00369452	54	35	5	0
qTL14b	*rpz4*	CI01000142_00459713_00463377	24	14	2	0
qBW4a	*gbp*	CI01000142_00593244_00613363	22	19	7	0
qBL9a	*lrp5*	CI01000153_00227118_00245589	77	65	8	0
qBW1a,qBL5a,qTL10a	*brca2*	CI01000095_00508378_00525085	116	39	36	2
qBW1a,qBL5a,qTL10a, qBL5b	*msi2*	CI01000093_00885214_00889462	18	42	0	1
	*lgals9*	CI01000093_01024056_01030224	38	21	6	0
qBW1b,qTL10b,qBH15a	*nos2b*	CI01000093_00515565_00527271	32	12	8	0
qBW1c,qBL5c,qBH15b	*mrps23*	CI01000093_00530430_00535255	52	24	2	0

## Discussion

### The colinearity of the genetic maps and the draft genome

The high-density linkage maps provide chromosomal frameworks for genome assembly validation. A total of 99 supercontigs with more than 20 SNPs were anchored onto the chromosomal framework. 45 of the anchored supercontigs were reversed in direction compared to other supercontigs. Most of the of supercontigs, were linear with the linkage group, but there were a few exceptions, such as the obvious scattering between LG12 ~ CI49 and CI50. This may be caused by the inaccuracies in the original sketch sequences, which need to be further refined to obtain more precise results. The LGs and supercontigs were not perfectly collinear if all markers are taken into analysis. The reason for this is hard to distinguish. This is because of the lack of parental linkage phase information, which can only be estimated by the offspring data with the introduction of some deviations. Therefore, the genetic linkage map is suitable for scaffold assembly and partial verification, and it is not suitable for the fragmented sequence assembly.

### The length of LG with different mapping algorithm and different gender

Pearson’s correlation coefficient among loci number, LG length and the average interval of three species -- grass carp, common carp and bighead carp were calculated (Table S4). The male, female and consensus map of grass carp were constructed through the ML algorithm and the regression algorithm was used in other fish. In the results, loci number and LG length had a strong correlation (≥0.92) in the ML maps, and the coefficients were generally around 0.6 in the regression maps (0.60 in common carp, 0.69 in bighead carp). This phenomenon may have been affected by the different mapping algorithms and it is indicated that in the ML maps more loci synchronized with the longer length of LGs.

Additionally, there were gender differences. In our ML maps, the loci of males were slightly less than that of females (1973 < 1989), but the total length of LGs was higher than that of females (6301.59 cM > 5680.51 cM). This was also found in the common carp with regression maps [14], which shows that this phenomenon was not caused by differences between mapping algorithms.

### The odd length of LG5

Notably, the length of LG5 is almost 3 times more than other LGs (Fig. 1). The LOD of markers of LG5 were more than 11.0, thus the effect of grouping error was excluded. The missing genotype of offspring in the SNPs would bring adverse effects to the accuracy of map distances, so the means of the missing genotypes in the markers on each LGs were calculated. The number for LG5 was 1.33, which was below the overall mean of 1.56. This indicated that LG5 abnormalities were not caused by a missing genotype.

In order to find out a reasonable explanation, all markers of LG5 were evaluated based on two versions of the grass carp genome: one was published online [23] and the other one was assembled by the PacBio sequences (unpublished data).

**Fig. 3:**
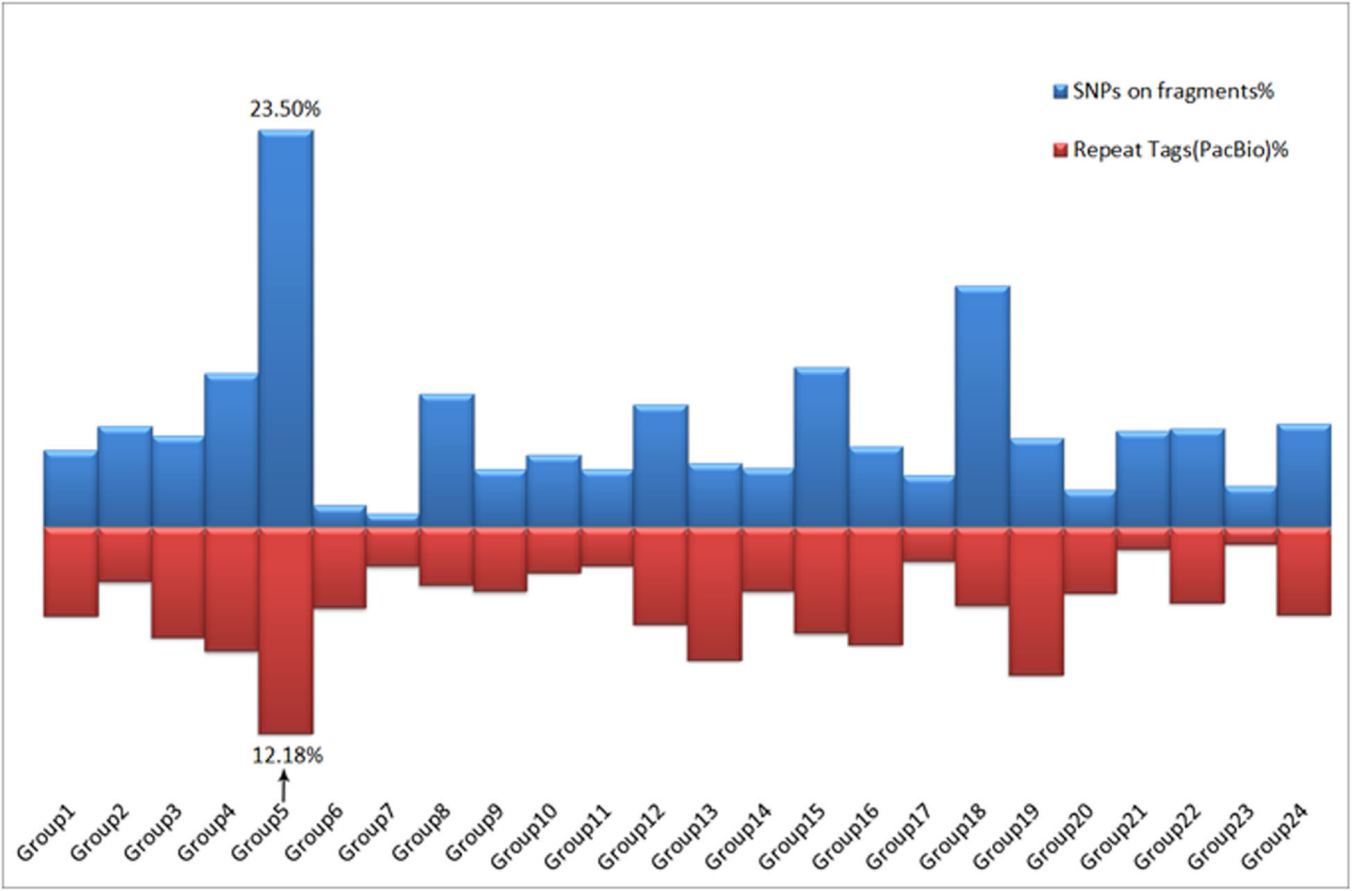
**The number of abnormal tags in each of the LGs.** The blue bar displayed the proportion of ‘fragment seq’ in each LGs. The red bar showed the proportion of repeat tags, which were detected through the PacBio contigs in each LGs

The published genome, N90 (179,941 bp) [23] was used as the standard for division. Then the 164,368 supercontigs were divided into two groups: ‘N90 seqs’ or ‘Fragment seqs’. It is well known that it is difficult to assemble sequences (fragment seqs) generally because of the repeated sequences, DNA secondary structure and other factors. The number of fragment seqs located in each LGs of the concensus map were counted (Fig. 3).

Moreover, 44 Gb of PacBio RSII sequences data were used for the de novo genome assembling. The sequencing sample was an adult grass carp, which was a third gynogenetic generation fish that had a nearly homozygous genome. The long sequencing reads and the homozygous genome made it easier to conquer the problem of the repeated regions than the published genome version. Then the markers on the map were mapped to the new de novo assembling contigs, the ‘new’ non-unique tags were counted (Fig. 3).

Judging from the statistical results, the percentage of SNP tags on either ‘fragments seqs’ or ‘repeated tags’ of LG5 were the highest. In summary, it was speculated that the length anomaly of LG5 was affected by more repeated tags. However, whether there were more recombination hot spots on LG5 that could not be determined.

### The growth-related QTL in the grass carp

QTL mapping is one of the important applications of genetic mapping. This is a bridge towards functional genome research from structural genome research and has important application value for production practice. Growth-related traits are the most important economic traits in aquaculture animals, as multiple genes, environments and their interactions control them. The current research on growth-related traits of QTL mapping is mainly concentrated on the Atlantic salmon, rainbow trout, perch, common carp and tilapia in Asia, the growth of grass carp QTL positioning characters are rarely reported. In this study, 30 growth-related QTLs were identified by analyzing 4 growth-related phenotype data and high-density genetic linkage maps of grass carp. For these locations, we found most of their locations were overlapped on linkage groups (Table 2), For example, marker ref-27,615 was located on LG18 and corresponded to 4 QTL, including qBW4a, qBL9a, qTL14b and qBH18. The reason for this result might be the high correlation coefficients among the 4 traits in grass carp. This can be seen in Table S2, the lowest correlation coefficients were 0.89 between BL and BH and the highest correlation coefficients were 0.97 between BL and TL.

### The candidate genes of the growth-related QTL

Due to differences in genomic structure, it is difficult to directly compare QTLs between species, while the comparison of homologous genes is feasible. However, we did not find any intersection between the candidate genes of grass carp and the genes of the growth-related QTLs reported in salmonid fishes [38]. A reasonable explanation is that a QTL analysis can only find a very limited set of genes and miss most growth-related genes, so it is unlikely that a gene will be repeatedly detected in different QTL studies. In addtion, we cannot rule out the possibility that different fish species (or even different parent of the same species) have their unique alleles leading to differential growth in offsprings.

The candidate gene list does not cover those well-known genes related to growth, such as growth hormone (GH) gene. This fact is likely to indicate that these essential growth-related genes are functionally conservative and structural mutations on them are rare in natural enviroment. However, some of the candidate genes in our research have been shown to be directly or indirectly related with the growth trait. For example, the gene rapunzel 4 (rpz4) on qTL14b was identified as the most significant QTL for TL in our research, its heterozygous missense mutation could result in axial skeletal overgrowth [39].

Another example came from Nitric Oxide Synthase (NOS). As a multifunctional messenger molecule, Nitric Oxide (NO) could be involved in neurogenesis, cell migration, immunity and apoptosis [40]. In zebrafish, Nitric Oxide Synthase 2 (NOS2) has two isoforms, NOS2a and NOS2b [41]. NOS2a is an innate immune factor and has been studied in mammals [42, 43] and fish [44, 45]. However, NOS2b was not localized in the immune cells during the development of embryos, and the result of whole-mount in site hybridization showed that it may play a role in neuropypophysis and thyroid primordium [41]. NOS2b protein in fish has a myristoylation consensus site at the extremity of the N-terminal, and is similar to mammal NOS3, which catalyze NO and mediates vascular endothelial growth factor (VEGF)-induced angiogenesis in coronary vessels [46]. All of these study are consistent to our results, in which NOS2b was significantly related to BW and BH.

## Conclusion

A high-density genetic linkage map of grass carp was built. The map’s correctness is supported by the good collinearity with both the grass carp draft genome and the zebrafish genome, and its effectiveness is demonstrated by the mounting rate which is much higher than the first map. A total of 30 growth related QTLs were detected, and 17 candidate genes were obtained from a cross analysis of the resequencing data from parent fishes, while the genes located on the QTLs without separable or effective SNPs were excluded.

## Methods

### The polymorphic SSRs genotyping of all parents

In order to select a suitable mapping population, the fin samples of 89 grass carp parents were captured from wild populations in the Yangtze River, Pearl River and Xiangjiang River. The samples were collected for genomic DNA extraction with the standard phenol-chloroform protocol [47]. Eighty-nine samples were genotyped by PCR with 11 SSR markers (Table S5).

The PCR reaction for each SSR was performed in 10 μL volumes containing 1 μL (about 20 ng) of sample DNA, 5 μL 2xEs Taq masteMix (CWBIO, CHINA), 0.1 μmol forward primer and 0.1 μmol reverse primer, under the following conditions: 94 °C 3 min, 35 cycles of 30 s at 94 °C, 30 s at 53 °C, 30 s at 72 °C, and then prolonged extension for 5 min at 72 °C. The PCR products were genotyped through ABI3730 (ABI, USA) and the matrix of PCR band sizes of the 89 samples on all 11 SSRs were obtained. The observed heterozygosity (Ho), expected heterozygosity (He) and polymorphism information content (PIC) of each SSR was calculated through cervus (v3.0.7) [34]. The average PIC of these markers was 0.84 and the minimum PIC was 0.78, indicating that these markers are highly polymorphic (Table 4).

**Table 4:** The genetic diversity of SSR markers

Locus	NO. of alleles	Hobs	HExp	PIC
G023	10	0.64	0.815	0.787
G5010	12	0.798	0.845	0.823
G5012	15	0.865	0.903	0.888
G5020	14	0.854	0.899	0.885
G5023	12	0.82	0.877	0.859
G5034	11	0.764	0.861	0.84
G5035	14	0.888	0.87	0.852
G5004	16	0.899	0.884	0.867
G5024	11	0.933	0.818	0.791
G5025	14	0.91	0.904	0.891
G5036	11	0.753	0.801	0.777

### Hierarchical cluster analysis of all parents

**Fig. 4:**
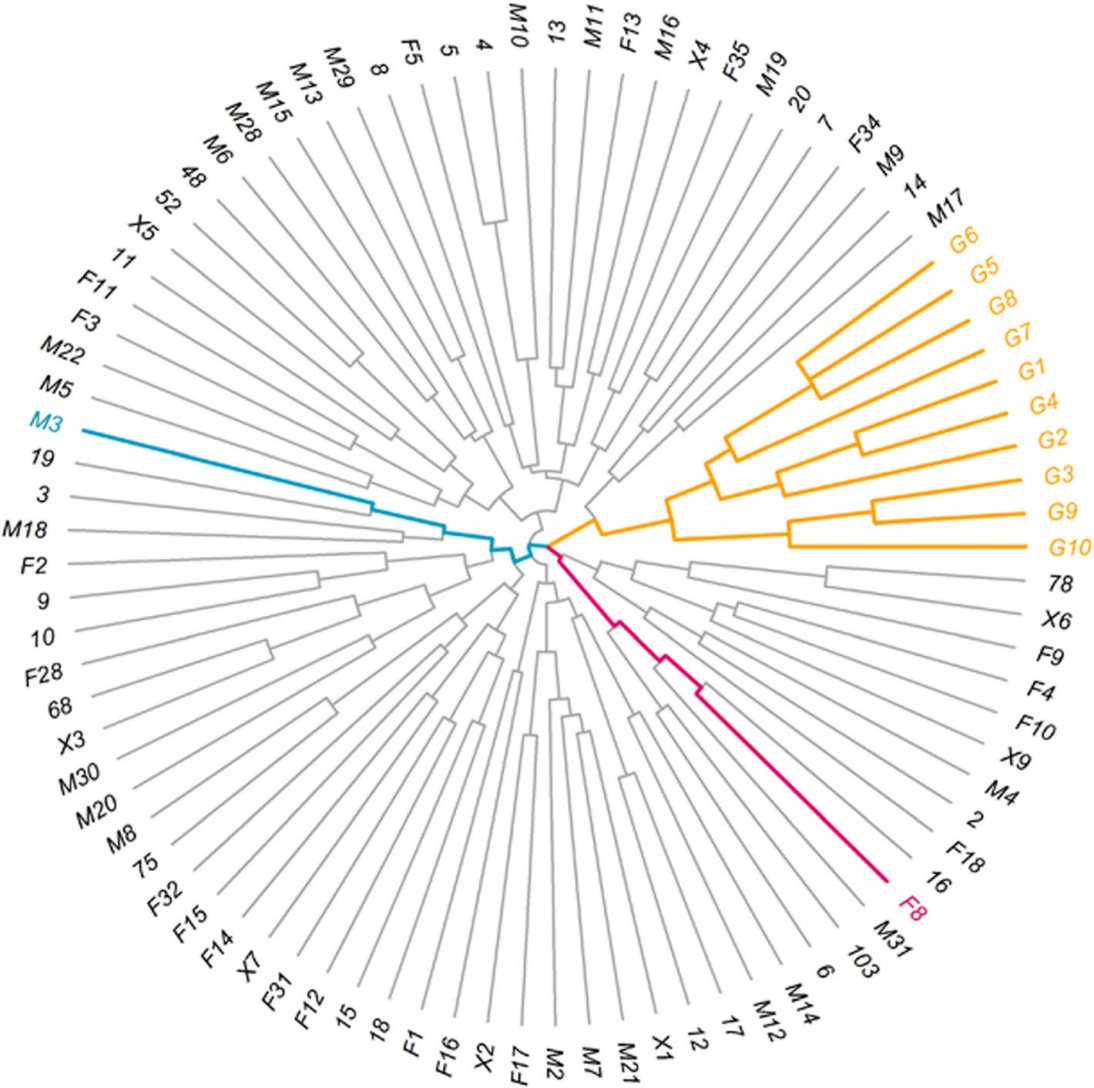
The hierachical clustering tree of the 89 grass carp parents based on SSR polymorphism. The orange branches were closely related fishes, the red branch was the mother (F8) and the blue branch was the father (M3)

Hierarchical clustering between samples was completed using R script. The band size was treated as a factor, rather than a numerical value. Between any two samples, the amount of different bands on every SSR loci were calculated as scores, then the Euclidean distance was calculated to determine the genomic similarity and the tree map was obtained (Fig. 4). Among all samples, G1-G10 were closely related and accurately clustered into a single branch. This supported the reliability of this method. Due to these results, male M3 from the Yangtze River and female F8 from the Pearl River were selected as the parents, and then their 100 randomly extracted progenies were used to construct the CP population.

### Mapping population and phenotypic data

The F1 progenies were bred in May 2015. 100 individuals that were 6 months old were randomly sampled. Growth-related traits, including body length (BL), total length (TL), body weight (BW) and body height (BH), were measured. The caudal fin of all samples including 89 adults and 100 progenies were preserved in 95% ethanol and the Genomic DNA was extracted following the standard phenol-chloroform protocol [47]. After sampling, all fish were released.

### 2b-RAD sequencing and screening of SNP tags

Libraries for 2b-RAD with BsaXI of two parents and 100 progenies were prepared [26] and then sequenced on X-10 (Illumina Inc.). Quality control was used in order to remove low-quality and non-restriction site tags (Table S6), then the parents’ data was mapped to the grass carp draft genome using SOAP2 with default values for all parameters. The tags which are uniquely mapped on the genome were filtered further by RADtyping to exclude those with a too high or too low sequencing depth [48]. After this process, the remaining unique reads from parent were used as the new reference sequences, to which all the data from progenies were mapped for genotyping. Markers were screened out as the preliminary SNP tags from the progenies’ reads by two criteria: (1) the markers were genotyped successfully in at least 80% of the progenies; (2) the markers were heterozygous in both of the parents.

In order to eliminate the errors which might be introduced into the uniqueness of markers by any single software, the preliminary SNP tags were mapped to the grass carp genome using bowtie (v1.2) [49] and bowtie 2 (v2.3.3) [50] respectively with default parameters, reads with more than 2 mismatches or Indels in any mapping were excluded. Finally, the alignment results were merged and significant segregation distortion markers were removed using χ^2^ test, and the final unique SNP tags were obtained. In addition, the *χ*^2^ test was done by R.

Considering that the length of linkage group 5 (LG5) is remarkably long, and in order to validate it, all tags were mapped to an upgraded grass carp genome, which was assembled with long reads (44×) generated by PacBio RSII.

### Construction of genetic maps and QTL mapping

To reduce calculation time and achieve the most accurate linkage results, SNPs with the same parental genotype, such as ‘ab x aa’, were used for determining whether they were completely linked or not. An in-house python script was written to complete this process, in which markers with missing genotypes in some of the fish offsprings are allowed.

The markers were grouped with a LOD threshold of 5.0 into 24 LGs using Joinmap 4.1 [51] with default parameters. The male and female maps were also calculated by Joinmap 4.1 with Monte Carlo ML algorithm [51] and the consensus map was merged by MergeMap [52] . Pearson’s correlations among the four growth-related traits (BW, BH, BL and TL) were performed in all progenies. QTL mapping for growth traits used the Multiple QTL Mapping (MQM) method, with a LOD interval of 1 cM through MapQTL 6.0 [53]. The consensus map and QTL mapping were visualized using circos (v0.66) [54].

### The synteny analysis of grass carp draft genome and zebrafish

After QTL mapping above, the correspondence of marker positions on the genetic map and draft genome were obtained. The supercontigs with more than 20 markers located on the genetic map were retained. The markers on these supercontigs were used for synteny analysis, and for visualizing the results of synteny analysis ggplot [55] was then used. For the sake of making the image aesthetically pleasing, the supercontigs were renamed. The corresponding supercontigs are listed in Table S1–7.

During the process of the synteny analysis of zebrafish, markers located on the grass carp genetic map were mapped to the zebrafish genome (GRCz10) using bowtie and bowtie 2 with default parameters to exclude repeated tags which can be mapped on multiple locations. Remaining unique SNP tags were used for the synteny analysis and the visualization of collinearity was done by circos (version: 0.66). The zebrafish chromosomal names were also renamed for aesthetic needs, and the corresponding list is shown in Table S2–7.

### Obtainment and analysis of parents resequencing data

After the DNA sequencing libraries were constructed with an insert size of 300 bp and paired-end sequenced on an Illumina Xten sequencer, the data of the parents was obtained. Then the filtered reads were mapped to the grass carp draft genome with BWA (version: 0.7.12) using the default parameters. Duplicated reads were filtered with Picard (version: 2.1.1). SNP and Indel calling was performed using the Genome Analysis Toolkit (GATK, version: 3.5) with the adjustment of parameter ‘-glm’.

The homozygous SNPs and Indels in both the parents were excluded firstly, and the rest of them were annotated with the gene transfer format (GTF) file of grass carp [20] and the SnpEff software [56] by using the default parameters. As the result of annotation, every SNP was assigned a label named as ‘effect impact’, which valued in a set of four ratings (High, Moderate, Low and Modifier), and can be used for subsequent filtering process. The interval size of upstream and downstream for each gene was 5 K in our analysis. Then the total SNPs which located in the gene-related regions, included upstream, 5’UTR, exon, intron, 3’UTR and downstream, were counted for each gene. We removed the markers that may have little effect on gene function with the ‘Modifier’ tags and definited the remaining SNPs and indels as the functional mutations. The number of functional SNPs/Indels per gene were calculated finally.

## Supplementary information

**Additional file 1.**

**Additional file 2.**

## Data Availability

The raw sequence data reported in this paper have been deposited in the Genome Sequence Archive [57] in BIG Data Center, Beijing Institute of Genomics (BIG), Chinese Academy of Sciences, and can be publicly downloaded under accession numbers PRJCA001074 (https://bigd.big.ac.cn/bioproject/browse/PRJCA001074) and PRJCA001162 (https://bigd.big.ac.cn/bioproject/browse/PRJCA001162).

## Abbreviation

SNPs: Single nucleotide polymorphisms
QTLs: Quantitative trait loci
NGS: Next-generation sequencing
CroPS: Complexity reduction of polymorphic sequences
RAD-seq: Restriction site associated DNA sequencing
MAS: Marker-assisted selection
Ho: Observed heterozygosity
He: Expected heterozygosity
PIC: Polymorphism information content
SSRs: Simple sequence repeats
BL: Body length
TL: Total length
BW: Body weight
BH: Body height
LG: Linkage group
GATK: Genome Analysis Toolkit
GTF: Gene transfer format
a-markers: Actual markers
GRs: Genome regions
GLRs: Genetic linkage regions
PVE: Phenotypic variance
GH: Growth hormone
NOS: Nitric Oxide Synthase
NO: Nitric Oxide
MQM: Multiple QTL Mapping
