# Supplementary material for: Construction of a high-density genetic map and mapping of growth related QTLs in the grass carp (Ctenopharyngodon idellus)
Source: BMC Genomics. 2020 Apr 19;21:313. doi: 10.1186/s12864-020-6730-x (PMC7168995; doi:10.1186/s12864-020-6730-x)
Supplement: Supplementary file 2 — Additional file 2. [file 12864_2020_6730_MOESM2_ESM.pdf]

Application Form for Laboratory Animal Welfare Ethic Review

(Institute of Hydrobiology, Chinese Academy of Sciences)

<table>
<tr><td colspan="3">Application No:　　　　　　　　　　　　　　　　Application date：2015/3/3</td></tr>
<tr><td colspan="3">Project name：Coupling of molecular modules for high-yield of carps</td></tr>
<tr><td colspan="3">Project source：Chinese Academy of Sciences　|　Project No.：XDA08020201</td></tr>
<tr><td colspan="3">Project name of the animal experiment：QTL analysis on growth trait of the grass carp</td></tr>
<tr><td>PI：Xiao-Qin Xia</td><td>Title：Professor</td><td>Tel：+86 27 68780915</td></tr>
<tr><td colspan="3">Experimenter：Xiaoli Huang</td></tr>
<tr><td colspan="3">Qualification of experimenter：confirmed</td></tr>
<tr><td rowspan="5">Information of animals</td><td colspan="2">Animal source：Huanggang Fisheries Research Institute</td></tr>
<tr><td>Species/strains：grass carp</td><td>Age or weight：
6-year-old parents and 6-month-old fingerlings</td></tr>
<tr><td colspan="2">Class：normal</td></tr>
<tr><td colspan="2">Number：189</td></tr>
<tr><td colspan="2">Planed experiment date：2015/5/5 to 2016/5/10</td></tr>
<tr><td colspan="3">Description of the animal experiment, incluing：objects, methods, measures, fate of animals, etc.

Object：
To locate the QTLs on genome related to the growth of grass carp

Methods and measures：
After anesthesia, a small amount of caudal fins will be cut from the individual, and the body length, width, and height of the individual were measured.

Animal fate:
After the collection, all individuals will be released back to the original fish pond without causing death.</td></tr>
</table>

Review points	1. Whether the project must use experimental animals for experiments, that is, whether it can use non-living methods such as computer simulation and cell culture to replace animals or lower animals to replace higher animals for experiments.<br>2. The applicant's qualifications listed in the table and the breeds, strains, quality levels, and specifications of the animals used are appropriate. Can the number of animals used be reduced by improving the design scheme or using high-quality animals.<br>3. Is it possible to optimize the experimental scheme and treat the animals well by improving the experimental methods, adjusting the experimental observation indicators, and improving the methods of sacrificing the animals.<br>4. Ensure the implementation of animal welfare measures.

Declaration：

I will strictly abide by the relevant regulations of animal welfare ethics of the People's Republic of China and Hubei Province, and accept the supervision and inspection of the Experimental Animal Ethics Committee of the Institute at any time.

PI: Xiaoqin Xia

Date： 2015.3.3

Comment:

I agree to approve this application.

Chairman of Ethic Committee for Laboratory Animal： [illegible]

date： 2015.3.3

Official seal:
